# A Case of Bullous Pemphigoid in a Five-Month-Old Female: A Rare Presentation

**DOI:** 10.7759/cureus.42871

**Published:** 2023-08-02

**Authors:** Mohamed Ahmed, Amani AlFalasi, Fatima M AlQaydi

**Affiliations:** 1 Dermatology, Dubai Academic Health Corporation, Dubai, ARE

**Keywords:** infantile bullous pemphigoid, skin blistering, bullous skin disease, vaccination, bullous pemphigoid

## Abstract

Bullous pemphigoid (BP) is the most common autoimmune subepidermal blistering disease, characterized by the presence of tense bullae and erosions of the skin and rarely at the mucous membranes. It often presents in the elderly population, but the incidence is rare in children and infants, with few reported cases in this population in the literature. We report a rare presentation of bullous pemphigoid in a five-month-old girl who presented to our clinic.

## Introduction

Bullous pemphigoid (BP) is an autoimmune subepidermal blistering disease, characterized by tense blisters, eosinophilia, and pruritus, and it often presents in the elderly population. It is rare in children and even rarer in infants [1,2]. The first case of BP in an infant was described in 1973. Since then, the number of reported cases has been increasing to reach 81 cases of BP in infants [3], 21 cases of which are related to vaccine administration [2]. Bullous pemphigoid during childhood has a benign, self-limited evolution, with lower recurrence rates and better prognosis when compared to the adult form [4,5].

We report a case of a five-month-old female infant who presented with bullous pemphigoid.

## Case presentation

**Figure 1:**
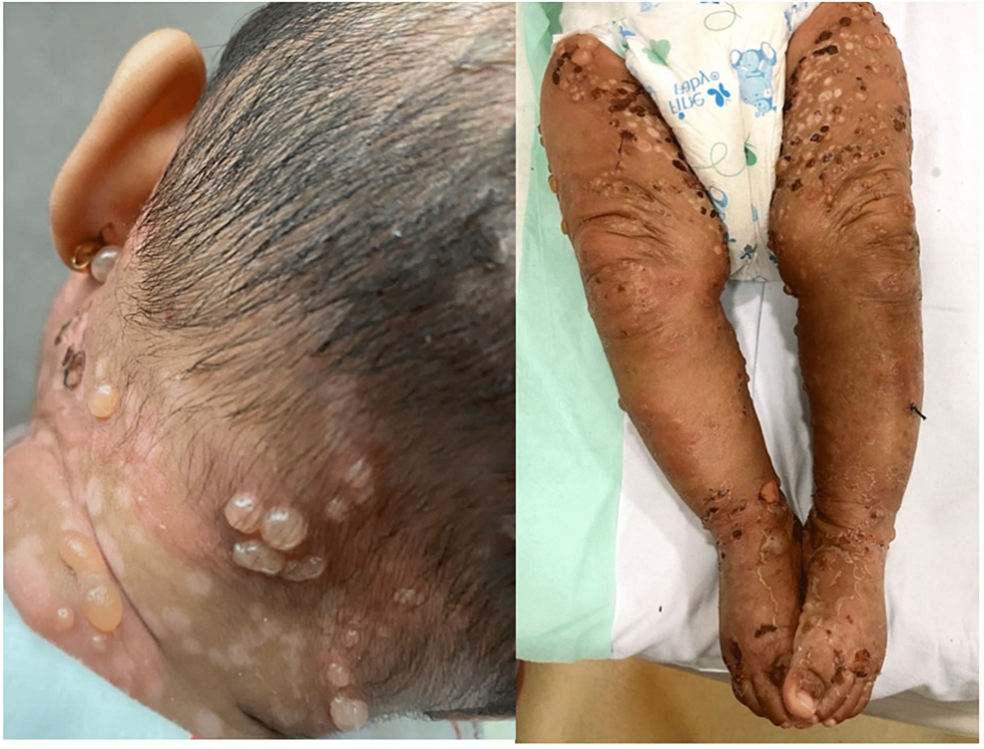
Blisters and bullae over the back of the scalp and neck, with some crusted erosions over the lower limbs

**Figure 2:**
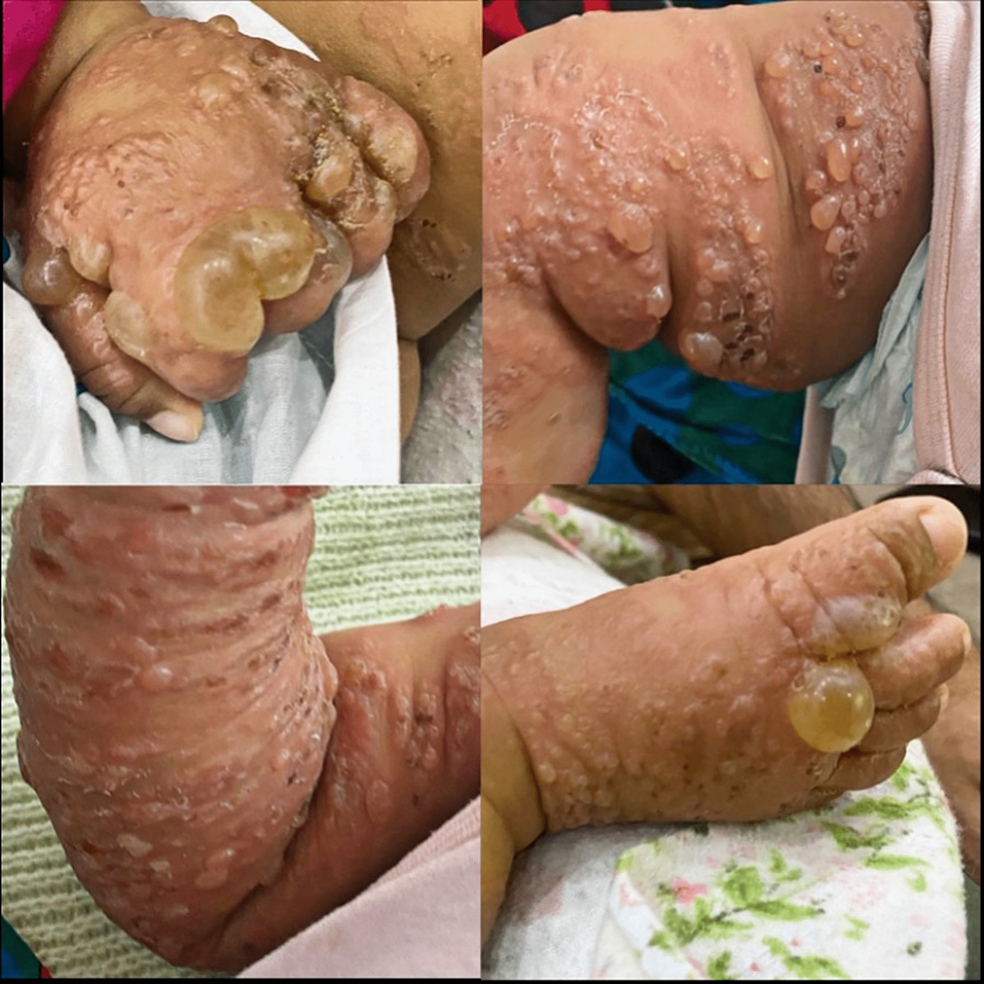
Multiple widespread firm vesicles, blisters, and bullae on the dorsal surfaces of the hands, thighs, arms, lower limbs, and feet

A previously healthy five-month-old girl presented to our dermatology center with the complaint of fluid-filled skin lesions over her limbs, trunk, and groin that developed over three weeks. She had originally been seen by her pediatrician, but as the blisters continued to develop, she was referred to us. The lesions were initially annular with erythematous borders and central clearing on palms and soles and gradually extended to involve the trunk, limbs, face, abdomen, back, and head. According to the parents, the child was feeding normally and denied having any fever or use of any medication. Their family history was unremarkable. The baby was born vaginally, post-term, without any complications. She received her second dose of DTaP (diphtheria, tetanus, and pertussis), Hib (Haemophilus influenzae type b), HBV (hepatitis B), pneumococcal, and oral polio vaccines two days prior to the onset of lesions. A dermatological examination revealed the presence of multiple widespread tense vesicles, bullae, and erosions located over the limbs, palms, soles, trunk, and face (Figures 1-2). There were also multiple annular urticarial erythematous plaques over the trunk. Oral and genital mucosae were not involved. Nikolsky sign was negative.

**Figure 3:**
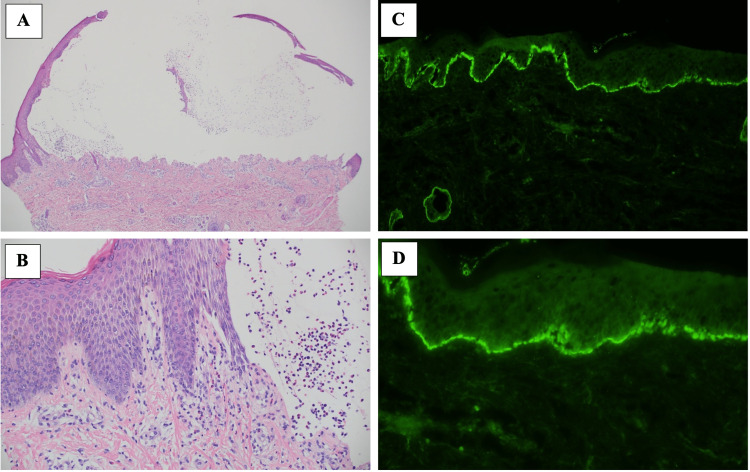
Histology shows (A) a subepidermal blister (HE stain, original magnification x40). (B) eosinophils infiltration (HE stain, original magnification x200). (C) direct immunofluorescence showing C3 deposition along the basement membrane zone. (D) direct immunofluorescence showing linear IgG along the dermo-epidermal junction IgG: immunoglobulin G

Complete blood count (CBC) showed leukocytosis (WBC: 17.82x10^3 uL) and eosinophilia (2178 cells/μL or 53% of total WBCs). A skin biopsy was performed, Histology revealed a subepidermal blister with eosinophils infiltration (Figure 3) and direct immunofluorescence (DIF) detected positive staining for linear immunoglobulin G (IgG) and C3 deposition in the epidermal basement membrane zone, staining for IgM and IgA are negative. The set of clinical findings and laboratory results confirmed the diagnosis of bullous pemphigoid.

**Figure 4:**
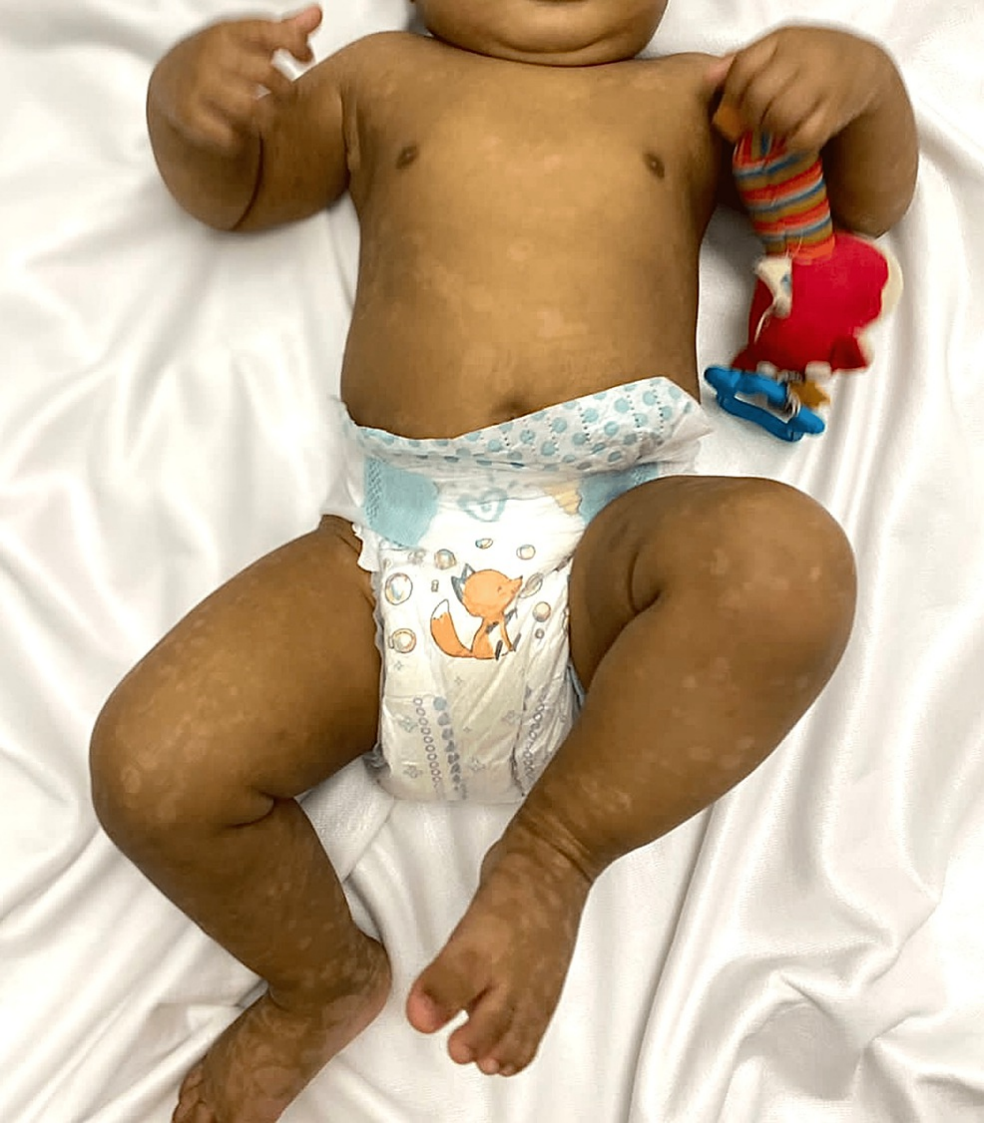
Complete resolution of the lesions, leaving hypopigmented macules

Treatment was started with oral prednisolone at a dose of 1 mg/kg and topical fusidic acid-betamethasone valerate 2-0.1 % cream daily. Reassessment after three weeks showed no obvious improvement, as erythematous plaques and new blisters continued to develop despite therapy. Therefore, oral dapsone (1.5 mg/kg/day) was added with rapid improvement, and the resolution of bullae was noticed. A glucose-6-phosphate dehydrogenase (G6PD) screen was done before starting dapsone, which was negative, and the complete blood count was normal without signs of anemia. After eight weeks of treatment, prednisolone was slowly tapered and eventually stopped. The patient showed complete resolution of the lesions (Figure 4) after 20 weeks, when dapsone was stopped.

## Discussion

Infantile BP is quite rare. To our knowledge, only 82 cases have been reported (including our patient) [3]. Compared to childhood bullous pemphigoid, infantile BP has more widespread clinical lesions with less mucosal involvement and predominant acral involvement [1,6]. In most cases, infantile BP has an excellent prognosis and responds well to topical and/or systemic corticosteroids. Some cases [1], including ours, were resistant to steroid treatment alone but responded well to the combination of dapsone and corticosteroids. Based on our study and other reports, dapsone is considered an effective, rapid, and safe treatment choice for steroid-resistant infantile BP. In our case, clinical improvement was noticed a few days after the introduction of dapsone, with no adverse effects observed in our patient.

In the minority of cases that were refractory, other agents were used, such as intravenous immunoglobulin (IVIG), mycophenolate mofetil, rituximab, or omalizumab, to control the disease [7]. Further studies are needed to reach a therapeutic ladder/consensus for infantile BP.

The possible relationship between vaccination and bullous pemphigoid has not been understood, as there is no clear evidence that vaccines are associated with an increased incidence of infantile BP. It could be a temporal relationship and not necessarily a causal relationship since many routine immunizations are performed within the first year of life [1]. More studies are required to rule out the association between BP and vaccinations. Healthcare providers have a crucial role in maintaining parents’ confidence in vaccines and maintaining the prevention of vaccine-preventable diseases.

## Conclusions

In conclusion, we describe a case of infantile BP successfully treated with the combination of dapsone and corticosteroid therapy, which could be considered as a possible treatment for patients resistant to conventional therapy. It is important for dermatologists to be aware of this clinical entity and to consider it among the differential diagnoses of vesiculo-bullous diseases in infants to allow early recognition and treatment of the disease.
